# Protein function prediction by collective classification with explicit and implicit edges in protein-protein interaction networks

**DOI:** 10.1186/1471-2105-14-S12-S4

**Published:** 2013-09-24

**Authors:** Wei Xiong, Hui Liu, Jihong Guan, Shuigeng Zhou

**Affiliations:** 1 School of Computer Science, and Shanghai Key Lab of Intelligent Information Processing, Fudan University, Shanghai, China; 2 Research Lab of Information Management, Changzhou University, Jiangsu, China; 3 Department of Computer Science & Technology, Tongji University, Shanghai, China

## Abstract

**Background:**

Protein function prediction is an important problem in the post-genomic era. Recent advances in experimental biology have enabled the production of vast amounts of protein-protein interaction (PPI) data. Thus, using PPI data to functionally annotate proteins has been extensively studied. However, most existing network-based approaches do not work well when annotation and interaction information is inadequate in the networks.

**Results:**

In this paper, we proposed a new method that combines PPI information and protein sequence information to boost the prediction performance based on collective classification. Our method divides function prediction into two phases: First, the original PPI network is enriched by adding a number of edges that are inferred from protein sequence information. We call the added edges implicit edges, and the existing ones explicit edges correspondingly. Second, a collective classification algorithm is employed on the new network to predict protein function.

**Conclusions:**

We conducted extensive experiments on two real, publicly available PPI datasets. Compared to four existing protein function prediction approaches, our method performs better in many situations, which shows that adding implicit edges can indeed improve the prediction performance. Furthermore, the experimental results also indicate that our method is significantly better than the compared approaches in sparsely-labeled networks, and it is robust to the change of the proportion of annotated proteins.

## Background

The past decade has witnessed a revolution in high-throughput sequencing techniques, resulting in huge amounts of sequenced proteins. However, experimental determination of protein functions is not only expensive but also time-consuming. As a consequence, there is an increasing concern about using computational methods to predict protein functions. Though many efforts have been made in this regard, the functions of most proteins in fully sequenced genomes still remain unknown. This is true even for the six well-studied model species. Taking yeast as an example, approximately one-fourth of the proteins have no annotated functions [1]. Therefore, functional annotation of proteins is one of the fundamental issues in the post-genomic era.

The most common approach to computational prediction of protein functions is to use sequence or structure similarity to transfer functional information among proteins. According to a recent survey [2], homology-based transfer approaches can be further divided into two classes: sequence-based approaches and structure-based approaches. BLAST [3] is one of the most widely used sequence-based approaches, which assigns un-annotated proteins with the functions of their homologous proteins. Although sequence similarity is undoubtedly correlated to functional similarity, in many cases there is no need to treat a protein as a whole, This is because typically only the 100-300 amino acids in a functional protein domain perform their functions [4]. Therefore, a protein can be represented as several sequence (or structure) based signatures (motifs) that are associated with some particular functions. PROSITE [5] for example is a database of sequence motifs that is composed of manually selected sequence motifs. Structure-based approaches are based on the observation that protein structure is far more conserved than sequence [6], and thus structure is a useful indicator of function. FATCAT [7] and PAST [8] are the most popular databases composed of 3D protein structures. The reason for using structure motifs is analogous to that of sequence motifs, One example is PROCAT [9], a library of 3D enzyme structure motifs. However, sequence similarity does not necessary imply functional equivalence and thus homology-based transfer approaches can result in erroneous predictions, and the original erroneous annotations may be propagated and amplified in databases [10]. Furthermore, as the databases expand, the utility of the homology-based transfer approaches begins to break down. For example, it has been estimated that < 35% of all proteins could be annotated automatically when accepting error rates of ≤ 5%, while even allowing for error rates of > 40%, there is no annotation for > 30% of all proteins [11].

Recent advances in experimental biology have enabled the production of vast amounts of protein-protein interaction (PPI) data across human and most model species. These data are commonly represented as networks, where a node corresponds to a protein and an edge corresponds to an interaction between a pair of proteins. Thus, using PPI data to assign protein function has been extensively studied. Approaches based on PPI data assume that proteins with similar functions are topologically close in the network. In a review of the existing computational approaches based on PPI data for protein function prediction, Sharan *et al. *[1] distinguished two types of approaches: direct annotation schemes and module-assisted schemes.

Direct annotation schemes predict the functions of a protein from the known functions of its neighbors, representatives are neighborhood counting approaches [12-15], graph theoretic approaches [16-18] and Markov random field (MRF) approaches [19-21]. *Majority *and *Indirect neighbors *are two neighborhood counting approaches. *Majority *[12] is the simplest direct approach, it utilizes the biological hypothesis that interacting proteins probably have similar functions, it ranks each candidate function based on its occurrences in the immediate neighbors. *Indirect neighbors *[13] assumes that proteins interact with the same proteins may also have some similar functions. It exploits both indirect and immediate neighbors to rank each candidate function. *Functional flow *[18] is a graph theoretic approach, it simulates a discrete-time flow of functions from all proteins. At every time step, the function weight transferred along an edge is proportional to the edge's weight and the direction of transfer is determined by the functional gradient. Deng *et al. *[19] devised an MRF model in which the function of a protein is independent of all other proteins given the functions of its immediate neighbors. The parameters of the model are first estimated using quasi-likelihood method, and then Gibbs sampling is used for inferring the functions of unannotated proteins.

Instead of predicting functions for individual proteins, module-assisted schemes first identify modules of related proteins and then annotate each module based on the known functions of its members, examples include hierarchical clustering-based approaches [22,23] and graph clustering approaches [24-27]. A key problem of this kind of approaches is how to define the similarity between two proteins. Arnau *et al. *[23] used the shortest path between proteins as a distance measure and applied hierarchical clustering to detecting functional modules. Up to now, numerous graph-clustering algorithms have been applied to detecting functional modules, such as spectral clustering [24], edge-betweenness clustering [25], clique percolation [26] and overlapping clustering [27].

Additionally, Chua *et al. *[28] presented a simple framework for integrating a large amount of diverse information for protein function prediction. This framework integrated diverse information using simple weighting strategies and a local prediction method. Hu *et al. *[29] hybridized the PPI information and the biochemical/physicochemical features of protein sequences to predict protein function. The prediction is carried out as follows: if the query protein has PPI information, the network-based method is applied; otherwise, the hybrid-property based method is employed.

However, most existing network-based approaches do not work well if there is not enough PPI information. In view of this, we proposed a new method that combines PPI information and protein sequence information to improve the prediction performance based on collective classification. Our method divided function prediction into two phases: First, the original PPI network is enriched by adding a number of edges that are computed based on protein sequence similarity. Second, based on the new network, a collective classification algorithm is employed to predict protein function. The main idea behind this method stems from the observation that existing network-based approaches ignore protein sequence information. Therefore, we increase the amount of useful information in the networks by adding a number of computed (or implicit) edges, which consequently improves the prediction performance.

We conducted experiments on *S.cerevisiae *and *M.musculus *functional annotation datasets. Compared to four existing protein function prediction methods, our method performs better in many situations, which shows that adding implicit edges can indeed improve the prediction performance. Furthermore, the experimental results also indicate that our method is significantly better than the compared methods in sparsely-labeled networks, and it is robust to the change of the proportion of annotated proteins.

## Methods

### Notation and problem definition

Protein function prediction is a multi-label classification problem where we have a set of functions $ℱ=\left(F_{1},\;\ldots ,\;F_{m}\right)$. Given a protein set, $Ƥ=\left(P_{1},\;\ldots ,\;P_{n}\right)$ where the first *l *proteins are labeled as *y*_1_, ..., *y_l_*, each *y_i _*is a vector with *y_ij _*= 1 in case that the protein *P_i _*is associated with the *j*-th function *F_j_*, otherwise *y_ij _*= 0. Our goal is to predict the labels *y*_1+1_, ..., *y_n _*for the remaining unlabeled proteins *P_l_*_+1_, ..., *P_n_*. In this study, we denote the PPI network as a finite undirected graph G=(ν,ε,W), with a vertex set ν=ℒ∪U where  ℒ corresponds to the set of annotated proteins and  U corresponds to the set of un-annotated proteins. Each edge *E_i_*_,*j*_∈ *ε* denotes an observed interaction between protein *V_i _*and *V_j _*and a weight *w_i,j _*∈ *W *indicates the interaction confidence between *V_i _*and *V_j_*. Here, we employ collective classification to tackle this problem. In addition, we use both explicit edges that are extracted from PPI datasets and implicit edges that are computed from protein sequence information. In what follows, we present the method in detail.

### Generating BLAST-inferred edges

As we pointed out above, most existing network-based approaches do not work well when there is not enough interaction information in the PPI networks. Considering this, here we propose a novel method that combines PPI information and protein sequence information to improve the prediction performance based on collective classification. The first step of our method is to enrich the original PPI network by adding a number of computed edges based on protein sequence similarity. Note that the similarity between two proteins is not a reliable proof that the two proteins interact, nevertheless, enriching PPI networks by adding a number of computed edges can increase the amount of useful information to the original PPI network and hence improve the prediction performance. In this paper, the basic local alignment search tool (BLAST) is employed to compute the similarity score between each pair of proteins.

For the protein *V_x_*, we define its sequence similarity scores with other proteins like this:

(1) $$S\left(V_{x}\right)=\left[s_{x,1},\;s_{x,2},\;\ldots ,\;s_{x,n}\right]$$

where *s_x,i _*denotes the similarity score between protein *V_x _*and protein *V_i_*. We set *s_x,i _*= 0 if *x *= *i*, which means that we do not consider self-similarity. For each protein *V_x _*in the original network, we create *k *edges to the *k *vertices that have the highest similarity scores with *V_x_*, and use the similarity scores as the weights of these created edges in the enriched network. Thus, we have

(2) $$S{\left(V_{x}\right)}_{topk}=\left[s_{x,1},\;s_{x,2},\;\ldots ,\;s_{x,k}\right].$$

It is worth noting that there are two types of edges in the new network: BLAST-inferred edges (*implicit edges*) and *explicit edges *that are already there. Here, two questions need to be answered. One is how many edges be added for each protein, that is, how to set the value of parameter k, and another is how to combine the weights of these two types of edges with different semantics. We will answer the first question in the experimental evaluation section and the second question in the next subsection.

### Gibbs sampling

The second step of our method is to employ the Gibbs sampling (GS) [30] based collective classification method o predict protein function based on the new network. GS is the most commonly used collective classification algorithm that aims at finding the best label estimate for each un-annotated vertex $V_{x}\in U$ by sampling each vertex label iteratively. GS based collective classification is divided into two phases: *boot-strapping *and *iterative classification*, its high-level pseudo-code is given in Algorithm 1. Detailed description on the algorithm is presented in the following subsections.

**Algorithm 1 **Gibbs sampling based collective classification for protein function prediction with implicit and explicit edges in PPI networks.

1:     // bootstrapping

2: **for **each query protein *V_x _***do**

3:         compute the initial $a_{x}^{\to }$ using $ℒ\cap N_{x}^{s}$ and $ℒ\cap N_{x}^{w}$

4: **end for**

5:     // burn-in period

6: **for ***i*=1 to *B *do

7:      **for **each query protein *V_x _***do**

8:             update $a_{x}^{\to }$ using current assignments to $N_{x}^{s}$, $N_{x}^{w}$

9:      **end for**

10: **end for**

11: // sampling period

12: **for ***i*=1 to *S ***do**

13:      **for **each query protein *V_x _***do**

14:             update $a_{x}^{\to }$ using current assignments to $N_{x}^{s}$, $N_{x}^{w}$

15:             create $b_{xi}^{\to }$ to record the *m*-rank result

16:      **end for**

17: **end for**

18: **for **each query protein *V_x _***do**

19:      calculate the final result $c_{x}^{\to }$ based on matrix *M_x_*

20: **end for**

#### Bootstrapping

According to the observation that proteins with shorter distance to each other in the network are more likely to have similar functions, weighted voting is employed to predict an initial functional probability distribution for the query protein. Note that there are two types of annotated neighbors to vote: implicit neighbors (BLAST-inferred neighbors) and explicit neighbors. Thus, we introduce a combination parameter λ ∈ (0, 1) to control the tradeoff between these two types of neighbors.

Formally, for a query protein *V_x _*that has *k *implicit neighbors and *N_x _*explicit neighbors, we define the corresponding edge weights like this:

(3) $$N_{x}^{s}=\left[s_{x,1},\;s_{x,2},\;\ldots ,\;s_{x,k}\right],\;\;N_{x}^{w}=\left[w_{x1},\;w_{x2},\;\ldots ,\;w_{xN_{x}}\right].$$

Above, $N_{x}^{s}$ and $N_{x}^{w}$ are the vectors of implicit edges and explicit edges, respectively. Then, the probability of *V_x _*associated with the *j*-th function *F_j _*is computed like this:

(4) $$P_{x}^{j}=\lambda \frac{1}{Z_{x}^{s}}\overset{k}{\underset{i=1}{\sum }}f_{i,j^{S}x,i}+\left(1-\lambda \right)\frac{1}{Z_{x}^{w}}\overset{N_{x}}{\underset{i=1}{\sum }}f_{i,j}w_{x,i}$$

where $Z_{x}^{s}$ and $Z_{x}^{w}$ are the normalizers:

(5) $$Z_{x}^{s}=\overset{k}{\underset{j=1}{\sum }}\overset{m}{\underset{i=1}{\sum }}f_{i,j^{S}x,i}\;Z_{x}^{w}=\overset{N_{x}}{\underset{j=1}{\sum }}\overset{m}{\underset{i=1}{\sum }}f_{i,j}w_{x,i}.$$

The larger the value of $P_{x}^{j}$, the more likely protein *V_x _*is associated with the *j*-th function *F_j_*. Given a query protein *V_x_*, its initial functional probability distribution is denoted as an m-dimensional vector:

(6) $$a_{x}^{\to }=\left[P_{x}^{1},\;P_{x}^{2},\;\ldots ,\;P_{x}^{m}\right].$$

Note that when predicting the functions of the query protein *V_x_*, we consider only its labeled neighbor proteins (either implicitly connected or explicitly connected). That is the reason why we use $ℒ\cap N_{x}^{s}$ and $ℒ\cap N_{x}^{w}$ in Algorithm 1 (Line 3), because unlabeled neighbor proteins can not be exploited in the bootstrapping phase. Codes corresponding to the *Bootstrapping *phase in Algorithm 1 are from Line 2 to Line 4.

#### Iterative classification

The iterative classification process is divided to the following two periods: the *burn-in *period and the *sampling *period. The *burn-in *period consists of a fixed number B of iterations where we update $a_{x}^{\to }$ using weighted voting. This period is implemented in Algorithm 1 from Line 6 to Line 10. The sampling period consists of *S *iterations. In each iteration, we not only update $a_{x}^{\to }$ but also maintain the count statistics as to how many times we have sampled the *j*-th function *F_j _*for protein *V_x_*. This period is implemented in Algorithm 1 from Line 12 to Line 20.

It is worth noting that most proteins in vivo often perform more than one function, thus, protein function prediction is a multi-label classification problem. For the query protein *V_x_*, its most likely function can be computed as follows:

(7) $$b_{x}^{1}=argmax_{j\in \left[1,m\right]}P_{x}^{j}$$

where $b_{x}^{1}$ represents the argument *j *that maximizes the value of $P_{x}^{j}$, which is regarded as the 1st-rank result. Accordingly, the second most likely function $b_{x}^{2}$ is regarded as the 2nd-rank result, and the third most likely function $b_{x}^{3}$ is regarded as the 3rd-rank result, and so forth. In rare case when more than one element $P_{x}^{j}$ in Eq. (7) has the same score, their ranks will be assigned randomly. So we can create an *m*-dimensional vector $b_{xi}^{\to }$ for the query protein *V_x _*to record its ranking result in the *i*-th iteration as follows:

(8) $$b_{xi}^{\to }=\left[b_{xi}^{1},\;b_{xi}^{2},\;\ldots ,\;b_{xi}^{m}\right].$$

When the threshold number *S *of iterations is reached, we can get a matrix *M_x _*with *S *rows and *m *columns for the query protein *V_x_*:

(9) $$M_{x}={\left[b_{x1}^{\to },\;b_{x2}^{\to }\;b_{xS}^{\to }\right]}^{T}.$$

In the first column of the matrix *M_x_*, the most frequently sampled function is denoted by $c_{x}^{1}$, called the first rank predicted function. Accordingly, in the second column of the matrix *M_x_*, the most frequently sampled function (excluding $c_{x}^{1}$) is denoted by $c_{x}^{2}$, called the second rank predicted function. In the third column of the matrix *M_x_*, the most frequently sampled function (excluding both $c_{x}^{1}$ and $c_{x}^{2}$) is denoted by $c_{x}^{3}$, called the third rank predicted function, and so forth. Finally, we get an *m*-dimensional vector $c_{x}^{\to }$ for the query protein *V_x_*:

(10) $$c_{x}^{\to }=\left[c_{x}^{1},\;c_{x}^{2},\;\ldots ,\;c_{x}^{m}\right].$$

## Results and discussion

### Interaction and annotation data

We evaluated the performance of our approach with two PPI datasets. The firs dataset (denoted as Dataset A) used in this study is based on Gene Ontology (GO) annotation scheme [31]. GO annotations are arranged in a hierarchical order, and consist of three basic GO namespaces: molecular function, biological process and cellular component. There are 19655 GO terms that constitute 15 levels of annotations, and the higher level terms are more generic while the lower level terms are more specific. In this setting, some vague terms such as "GO:0005554 molecular function unknown" and annotations with evidence code "IEA" (Inferred from Electronic Annotation) were excluded. Furthermore, to avoid the bias problem in the annotations, we applied the concept of informative Functional Class [13] to selectively identify GO terms for validation. An informative GO is referred as the one that 1) is annotated by at least 30 proteins; and 2) has no child terms annotated by at least 30 proteins. This ensures that terms used for validation have a reasonable number of annotations and do not have overlapping description. Predictions were performed separately for each namespace. As a result, in the *S.cerevisiae *annotation dataset, there are 39, 95 and 66 informative GO terms and in the *M.musculus *annotation dataset, there are 103, 334 and 130 informative GO terms for the molecular function, biological process and cellular component namespaces, respectively.

Protein interactions of Dataset A were downloaded from the Biological General Repository for Interaction Datasets (BioGRID) [32]. BioGRID is a public database that archives and disseminates genetic and protein interaction data from model organisms and humans, it currently holds 347966 interactions (170162 genetic, 177804 physical) obtained from both high-throughput data sets and individual focused studies, which were derived from over 23000 publications in the literature.

In this study, we constructed one protein interaction network for each GO namespace using only physical interactions. Therefore, there are totally six PPI networks (three for *S.cerevisiae *and the other three for *M.musculus*) in Dataset A. In these networks, a node corresponds to a protein and a un-weighted edge corresponds to an interaction between two proteins. Each node was assigned with at least one Go term, and proteins without interaction data or sequence information were deleted. The details for these networks are shown in Table 1.

**Table 1:** Statistics for Dataset A.

Namespaces	S.cerevisiae proteins	S.cerevisiae interactions	M.musculus proteins	M.musculus interactions
				
molecular function	1147	20013	715	1855
biological process	3277	54983	1046	2729
cellular component	4497	74422	1406	3614

The second dataset (denoted as Dataset B) used in this study is based on Functional Catalogue (Fun-Cat) annotation scheme [33] taken from Munich Information Center for Protein Sequences (MIPS) (http://www.helmholtz-muenchen.de/en/ibis). Fun-Cat is organized as a hierarchically structured annotation system and consists of 28 main functional categories. FunCat annotations for *S.cerevisiae *were downloaded from Comprehensive Yeast Genome Database (CYGD) [34]. CYGD is a frequently used public resource for yeast related information. There are totally 6168 proteins in the dataset, of which 4774 were annotated. These proteins belong to 17 functional categories. The number of proteins for each functional category is listed in Table 2. FunCat annotations for *M.musculus *were downloaded from Mouse functional Genome Database (MfunGD) [35]. MfunGD provides a resource for annotated mouse proteins and comprises 17643 annotated proteins. These annotated proteins belong to 24 functional categories, which are also shown in Table 2.

**Table 2:** Statistics for Dataset B

MIPS Functional Category		CYGD	MfunGD
01	METABOLISM	942	2662
02	ENERGY	151	603
04	STORAGE PROTEIN	0	0
10	CELL CYCLE AND DNA PROCESSING	1010	1113
11	TRANSCRIPTION	1078	2119
12	PROTEIN SYNTHESIS	480	490
14	PROTEIN FATE (folding, modification, destination)	1155	2484
16	PROTEIN WITH BINDING FUNCTION OR COFACTOR REQUIREMENT (structural or catalytic)	1049	8369
18	REGULATION OF METABOLISM AND PROTEIN FUNCTION	249	1112
20	CELLULAR TRANSPORT, TRANSPORT FACILITIES AND TRANSPORT ROUTES	1042	2407
30	CELLULAR COMMUNICATION/SIGNAL TRANSDUCTION MECHANISM	234	4061
32	CELL RESCUE, DEFENSE AND VIRULENCE	554	769
34	INTERACTION WITH THE ENVIRONMENT	463	1486
36	SYSTEMIC INTERACTION WITH THE ENVIRONMENT	0	2073
38	TRANSPOSABLE ELEMENTS, VIRAL AND PLASMID PROTEINS	120	11
40	CELL FATE	273	1312
41	DEVELOPMENT (Systemic)	69	1042
42	BIOGENESIS OF CELLULAR COMPONENTS	863	979
43	CELL TYPE DIFFERENTIATION	452	370
45	TISSUE DIFFERENTIATION	0	426
47	ORGAN DIFFERENTIATION	0	559
70	SUBCELLULAR LOCALIZATION	0	9739
73	CELL TYPE LOCALIZATION	0	273
75	TISSUE LOCALIZATION	0	366
77	ORGAN LOCALIZATION	0	619

Protein interactions of Dataset B were downloaded from STRING database [36], which is an integrated protein interaction database containing known and predicted protein interactions. These interactions were mainly derived from four data sources: genomic context, high-throughput experiments, conserved co-expression and previous knowledge. The most recent version of STRING covers about 5.2 million proteins from 1133 organisms. For Dataset B, we constructed two PPI networks (one for *S.cerevisiae *and another for *M.musculus*), proteins without interaction data or sequence information were deleted. As a result, in the *S.cerevisiae *interaction network, there are totally 388846 distinct interactions among 4687 proteins, and in the *M.musculus *interaction network there are 14269 proteins and 832124 interactions. Additionally, protein sequence information for Dataset A and Dataset B were also downloaded from the STRING database.

### Competing approaches

We compared our method with a sequence similarity based approach (termed BLAST-mined) that does not take the PPI network into account. The BLAST-mined approach was performed in two steps. First, BLAST was adopted to compute similarity score between each pair of proteins. Second, we employed the *k*NN classifier to predict the functions of un-annotated proteins. We also conducted comparison with a graph based method: *Functional flow*, as well as two neighbor counting methods: *Majority *and *Indirect neighbors*. *Functional flow *[18] treats each annotated protein as the source of a functional flow. After simulating the spread over time of this functional flow through the network, each un-annotated protein is assigned a score for having the function based on the amount of flow it received during the simulation. *Majority *[12] makes use of the observation that interacting proteins are more likely to have similar functions, it determines the functions of a protein based on the known functions of proteins lying in its immediate neighborhood. The principal advantages of the *Majority *are its simplicity and effectiveness. *Indirect neighbors *[13] exploits both direct and indirect function associations. It computes scores based on level 1 and level 2 interaction partners of a protein.

### Experimental setup

For traditional classification problems, the standard evaluation criterion is accuracy. However, in this paper we can not simply determine whether a prediction is correct or wrong because of the partially correct phenomenon in multi-label classification problems [37]. Therefore, as in [38] we adopted the widely-used performance measure, the ratio of *TP*/*FP*, which depicts the relative magnitude between the number of true positives and the number of false positives. In this setup, we define the *i*-th rank overall *true positive *(*TP*) as the number of proteins whose *i*-th rank predicted function $c_{x}^{i}$ is one of the true functions of the protein *V_x _*and the *i*-th rank overall *false positive *(*FP*) as the number of proteins whose *i*-th rank predicted function $c_{x}^{i}$ is not one of the true functions of the protein *V_x_*. To evaluate the prediction performance of our method, leave-one-out cross validation was used to compare the performance of our method with that of the competing approaches. The idea behind leave-one-out cross validation is simply to treat each annotated protein as un-annotated in turn, then run the algorithm and compare the predicted functions to the known functions of the protein. It is worth noting that the iterative classification step is omitted in leave-one-out validation, this is because the label vector of the query protein is never updated after bootstrapping. However, in real PPI networks, there are a significant number of un-annotated proteins, thus leave-one-out cross validation seems impracticable in reality. Therefore, we also compared the performance of our method with that of the competing approaches in sparsely-labeled networks. In our implementation, the proportion of annotated proteins was varied from 10% to 90%, we ran 10 experiments for each given proportion of annotated proteins and reported the average performance. Moreover, the burn-in period and the sampling period were set to contain 20 and 100 iterations respectively.

### Effect of parameters **λ **and ***k***

We studied the effect of two parameters used in our study. The first one is the combination parameter λ that controls the tradeoff between implicit (BLAST-inferred) edges and explicit edges. We varied λ from 0.1 to 0.9 and compared the prediction performance. Table 3 and Table 4 lists the performance of different λ in dataset A, and Table 5 details the performance of different λ in dataset B. The experimental results show that the prediction performance is not definitely sensitive to the value of λ, as long as it is not chosen extremely small or extremely large. Thus, in our following experiments, the value of λ was set to 0.3 for both datasets. Next, we examined the effect of the number of BLAST-inferred edges *k*. We varied k from 1 to 15 for BLAST and compared the prediction performance. Table 6 and Table 7 gives the performance of different *k *in dataset A, and Table 8 shows the performance of different *k *in dataset B. We can see that when using *k*=5 for both dataset A and dataset B the proposed method performs best. This is because adding BLAST-inferred edges with low sequence similarity (when *k *is large) may produce false predictions. Hence, in our rest experiments, the value of k was set to 5.

**Table 3:** The effect of the combination parameter *λ *(Dataset A: S.cerevisiae)

parameter λ	molecular function		biological process		cellular component	
	
	1st	2nd	1st	2nd	1st	2nd
**Origin**	**1.083**	**0.136**	**0.587**	**0.190**	**0.923**	**0.408**

λ=0.1	1.381	0.197	0.648	0.201	0.983	0.429
λ=0.3	**1.703**	0.250	**0.818**	**0.239**	**1.174**	**0.493**
λ=0.5	1.630	**0.267**	0.781	0.223	1.133	0.407
λ=0.7	1.513	0.226	0.692	0.210	1.053	0.445
λ=0.9	1.273	0.178	0.617	0.195	0.873	0.378

**Table 4:** The effect of the combination parameter *λ *(Dataset A: M.musculus)

parameter λ	molecular function				biological process			cellular component		
	
	1st	2nd	3rd	1st	2nd	3rd	4th	1st	2nd	3rd
**Origin**	**0.282**	**0.124**	**0.099**	**0.389**	**0.233**	**0.127**	**0.087**	**1.632**	**0.449**	**0.237**

λ=0.1	0.327	0.160	0.149	0.416	0.229	0.131	0.104	1.762	0.549	0.267
λ=0.3	**0.470**	0.285	**0.230**	**0.563**	**0.317**	0.157	0.135	**2.020**	**0.670**	**0.319**
λ=0.5	0.442	**0.296**	0.161	0.537	0.267	0.147	**0.146**	1.850	0.618	0.279
λ=0.7	0.404	0.253	0.183	0.493	0.285	**0.168**	0.109	1.654	0.540	0.245
λ=0.9	0.376	0.206	0.110	0.429	0.243	0.115	0.131	1.522	0.414	0.197

**Table 5:** The effect of the combination parameter *λ *(Dataset B).

parameter λ	S.cerevisiae			M.musculus		
	
	1st	2nd	3rd	1st	2nd	3rd
**Origin**	**2.226**	**0.754**	**0.493**	**1.941**	**1.488**	**0.818**

λ=0.1	2.355	0.788	0.557	2.115	1.512	0.851
λ=0.3	**2.833**	**0.815**	0.639	**2.446**	**1.632**	**0.873**
λ=0.5	2.560	0.752	**0.663**	2.343	1.369	0.820
λ=0.7	1.893	0.695	0.580	2.011	1.440	0.775
λ=0.9	1.564	0.629	0.413	1.761	1.276	0.724

**Table 6:** The effect of the number of BLAST-inferred edges *k *(Dataset A: S.cerevisiae)

parameter *k*	molecular function		biological process		cellular component	
	
	1st	2nd	1st	2nd	1st	2nd
**Origin**	**1.083**	**0.136**	**0.587**	**0.190**	**0.923**	**0.408**

*k*=1	1.439	0.219	0.689	0.190	1.010	0.429
*k*=5	**1.703**	0.250	**0.818**	**0.239**	1.174	**0.493**
*k *=10	1.630	**0.265**	0.786	0.206	**1.225**	0.467
*k *=15	1.615	0.239	0.754	0.224	1.130	0.449

**Table 7:** The effect of the number of BLAST-inferred edges *k *(Dataset A: M.musculus)

parameter *k*	molecular function				biological process			cellular component		
	
	1st	2nd	3rd	1st	2nd	3rd	4th	1st	2nd	3rd
**Origin**	**0.282**	**0.124**	**0.099**	**0.389**	**0.233**	**0.127**	**0.087**	**1.632**	**0.449**	**0.237**

*k*=1	0.391	0.237	0.150	0.470	0.255	0.147	0.115	1.708	0.497	0.270
*k*=5	0.470	**0.285**	**0.230**	**0.563**	0.297	**0.177**	**0.135**	**2.020**	**0.670**	0.319
*k*=10	**0.491**	0.242	0.212	0.538	0.316	0.142	0.109	1.943	0.579	0.293
*k*=15	0.456	0.266	0.203	0.493	**0.339**	0.151	0.123	1.857	0.620	**0.331**

**Table 8:** The effect of the number of BLAST-inferred edges k (Dataset B).

parameter *k*	S.cerevisiae			M.musculus		
	
	1st	2nd	3rd	1st	2nd	3rd
**Origin**	**2.226**	**0.754**	**0.493**	**1.941**	**1.488**	**0.818**

*k*=1	2.112	0.636	0.446	2.153	1.510	0.823
*k*=5	**2.833**	**0.815**	0.639	**2.446**	**1.632**	**0.873**
*k*=10	2.572	0.754	**0.651**	2.330	1.565	0.845
*k*=15	2.395	0.692	0.616	2.275	1.493	0.757

### Leave-one-out cross validation experiments

**Figure 1:**
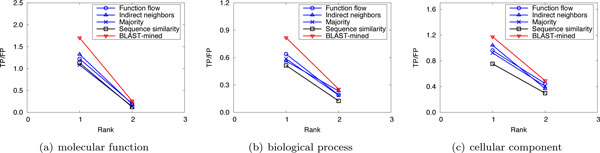
**Performance comparison by leave-one-out validation (Dataset A: S.cerevisiae)**.

**Figure 2:**
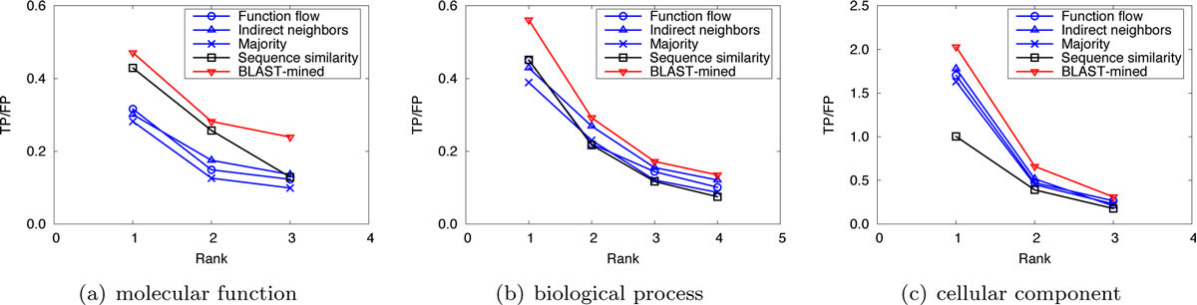
**Performance comparison by leave-one-out validation (Dataset A: M.musculus)**.

**Figure 3:**
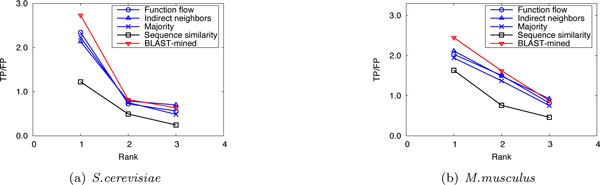
**Performance comparison by leave-one-out validation (Dataset B)**.

To evaluate the prediction performance of our method, leave-one-out cross validation was used to compare the performance of our method with that of the competing approaches. For *S.cerevisiae *in Dataset A, there are three PPI networks (corresponding to the three GO namespaces). The average number of functions that each protein has in these networks is 1.24, 1.33 and 1.64, respectively. So we considered only the top 2 (⌊1.24⌋ + 1, ⌊1.33⌋ + 1 and ⌊1.64⌋ + 1 are all 2) predictions. Figure 1 shows the performance comparison of our approach to the competing methods for the top-2 predictions. For *M.musculus *in Dataset A, as the average number of functions that a protein possesses in the three original networks is 2.16, 3.96 and 2.79, we considered only the first 3, 4, and 3 predictions, respectively. The results are shown in Figure 2. In Dataset B, there are two PPI networks (corresponding to S.cerevisiae and M.musculus). The average number of functions that each protein has in these networks is 2.13 and 2.58, so we considered only the top 3 ranks. The results are shown in Figure 3. It can be seen from Figure 1, 2, 3 that our method can obtain more accurate predictions than the four competing approaches, due to the combination of implicit (BLAST-inferred) and explicit edges. These results indicate that enriching PPI networks by adding a number of BLAST-inferred edges can indeed improve prediction performance. The experimental results also validate that the network-based approaches outperform the sequence similarity based approach in most cases. This is because the sequence similarity based approach is completely based on protein sequence information, and thus perform the worst. In addition, it is worth noting that higher rank functions are predicted better than lower ones, implying that the protein functions are well ranked by our approach.

### Performance in sparsely-labeled networks

**Figure 4:**
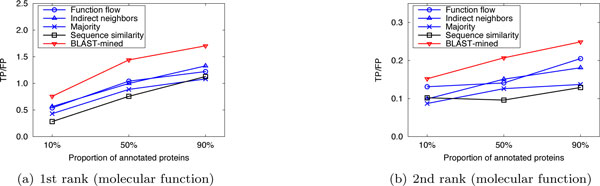
**Performance comparison in sparsely-labeled networks (Dataset A: S.cerevisiae)**.

**Figure 5:**
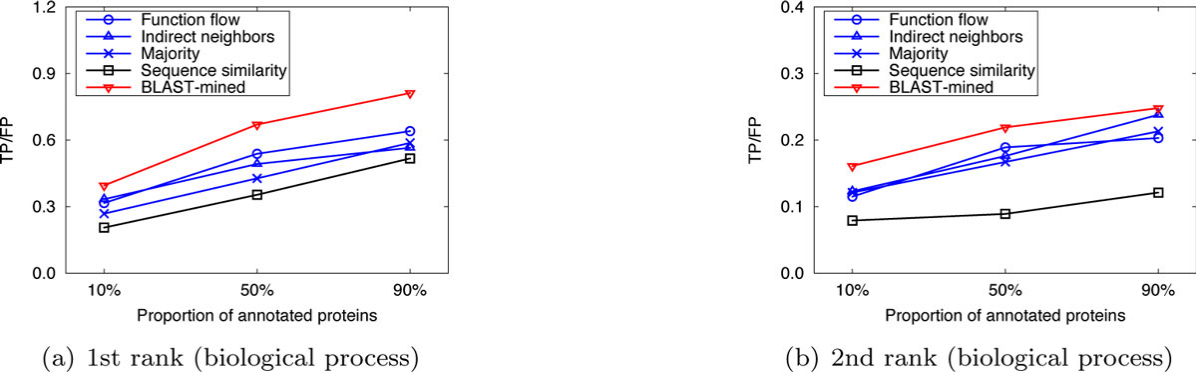
**Performance comparison in sparsely-labeled networks (Dataset A: S.cerevisiae)**.

**Figure 6:**
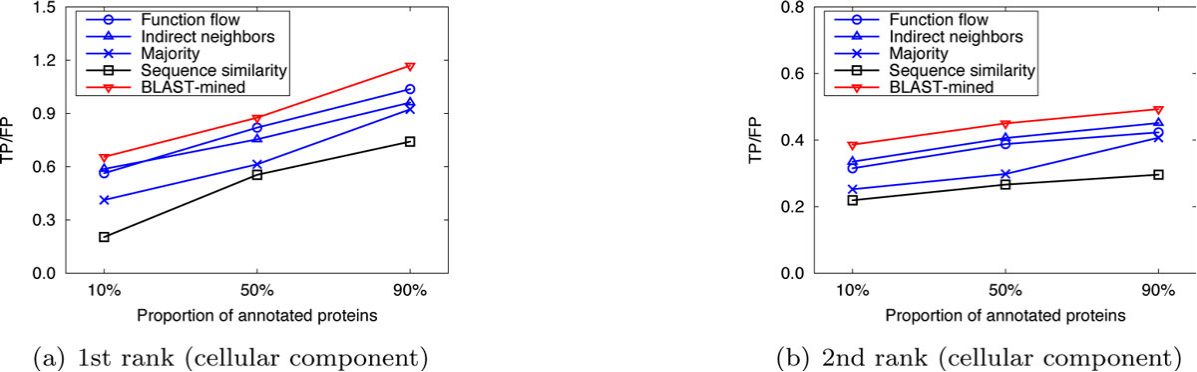
**Performance comparison in sparsely-labeled networks (Dataset A: S.cerevisiae)**.

**Figure 7:**
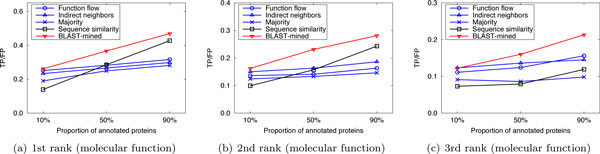
**Performance comparison in sparsely-labeled networks (Dataset A: M.musculus)**.

**Figure 8:**
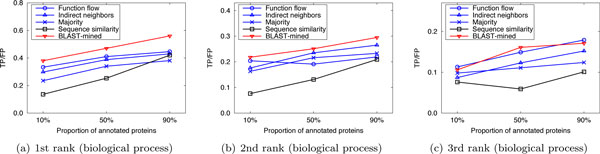
**Performance comparison in sparsely-labeled networks (Dataset A: M.musculus)**.

**Figure 9:**
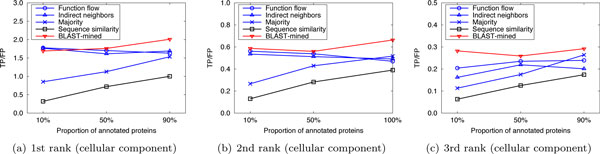
**Performance comparison in sparsely-labeled networks (Dataset A: M.musculus)**.

**Figure 10:**
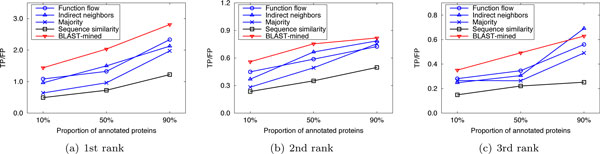
**Performance comparison in sparsely-labeled networks (Dataset B: S.cerevisiae)**.

**Figure 11:**
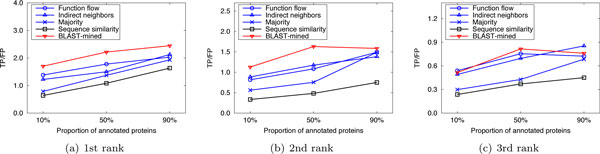
**Performance comparison in sparsely-labeled networks (Dataset B: M.musculus)**.

Here we compared the performance of our method with that of the competing approaches in sparsely-labeled networks. In our implementation, the proportion of annotated proteins in PPI networks was varied from 10% to 90%, with which we predicted the functions of the remaining (un-annotated) proteins. We ran 10 experiments for each given proportion of annotated proteins and evaluated the average performance. Figure 4, 5, 6 and Figure 7, 8, 9 present the results over *S.cerevisiae *and *M.musculus *data in Dataset A, and Figure 10 and Figure 11 show the results over the *S.cerevisiae *and *M.musculus *data in Dataset B. These results clearly show that our method performs better than the four compared approaches in most cases. The experimental results also validate that generally for all approaches the prediction performance gets better as the number of annotated proteins in the network increases. However, very interestingly we noticed that in Figure 9, the prediction performance of Function flow and Indirect neighbors slightly degrade as the number of annotated proteins in the network increases. And in Figure 11, when the ratio of annotated proteins increases up to 50%, prediction performance of our approach (for the 2nd and 3rd rank functions) turns slightly down. This may be due to the overfitting effect or annotation quality problem. Specifically, when the proportion of annotated proteins is 90%, the predicted functions of the un-annotated proteins are mainly based on the immediate neighbors, annotation quality will considerably impact the prediction performance. However, when the ratio of annotated proteins is 10%, the predicted functions of the un-annotated proteins are mainly based on the whole network, which thus alleviates the impact of annotation quality.

## Conclusion

In this paper, we proposed a new method to protein function prediction that combines PPI information and protein sequence information to improve prediction performance. It first reconstructs PPI networks by adding a number of BLAST-inferred implicit edges, and then applies the collective classification method to predicting protein functions based on the new networks. The key idea of our work is to enrich the PPI information of PPI networks by adding a number of computed edges, which subsequently improves the prediction performance. We carried out experiments on *S.cerevisiae *and *M.musculus *functional annotation datasets. The experimental results demonstrate that our method outperforms the existing approaches across a series of label situations, especially in sparsely-labeled networks where the existing approaches do not work well due to PPI information inadequacy. Experimental results also validate the robustness of the proposed approach to the number of labeled proteins in PPI networks.

In this paper, we used a very simple scheme (BLAST alignment) to infer implicit edges. Actually, there are some other methods that can be used to mine useful implicit edges, such as random walk. Random walk exploits both local and global network information, should be able to discover more useful hidden edges. We will explore this direction in the future.

## Competing interests

The authors declare that they have no competing interests.

## Authors' contributions

Shuigeng Zhou and Jihong Guan conceived the study, and revised the manuscript. Wei Xiong performed all experiments and data analysis, and drafted the manuscript. Hui Liu prepared the datasets.

## Declarations

The publication costs for this article were funded by the corresponding author.

This article has been published as part of BMC Bioinformatics Volume 14 Supplement 12, 2013: Selected articles from the IEEE International Conference on Bioinformatics and Biomedicine 2012: Bioinformatics. The full contents of the supplement are available online at http://www.biomedcentral.com/bmcbioinformatics/supplements/14/S12.
